# Supplementary material for: Incidence and predictors of antiretroviral treatment failure among children in public health facilities of Kolfe Keranyo Sub-City, Addis Ababa, Ethiopia: Institution-based retrospective cohort study
Source: PLoS One. 2023 Aug 18;18(8):e0266580. doi: 10.1371/journal.pone.0266580 (PMC10437829; doi:10.1371/journal.pone.0266580)
Supplement: Attachment — Submitted filename: PONE-D-22-0848withcomments7.pdf [file pone.0266580.s002.pdf]

PLOS ONE

Incidence and Predictors of Antiretroviral Treatment Failure among Children in Public Health Facilities of Kolfe Keranyo Sub-City, Addis Ababa, Ethiopia: Institution-based retrospective cohort study

--Manuscript Draft--

**Manuscript Number:**	PONE-D-22-08487
**Article Type:**	Research Article
**Full Title:**	Incidence and Predictors of Antiretroviral Treatment Failure among Children in Public Health Facilities of Kolfe Keranyo Sub-City, Addis Ababa, Ethiopia: Institution-based retrospective cohort study
**Short Title:**	Incidence and Predictors of Antiretroviral Treatment Failure among Children
**Corresponding Author:**	Meseret Misasew, MPH<br>CDC: Centers for Disease Control and Prevention<br>Addis Ababa, Adis Abeba ETHIOPIA
**Keywords:**	ART, Treatment failure, Children, Incidence, Addis Ababa.
**Abstract:**	Abstract<br><br>Background : Human immunodeficiency virus (HIV) infection is a public health concern globally. The numbers of people living with HIV worldwide in 2018 was estimated at 37.9 million; from those, 1.7 million are children. Globally, 62% of the 37.9 million people were receiving Antiretroviral treatment (ART); 53% had achieved viral suppression. This study aimed to assess the incidence and predictors of Antiretroviral treatment failure among children in Kolfe Keranyo sub-city, Addis Ababa, Ethiopia.<br><br>Methods : An institution-based retrospective cohort study was conducted among 250 children who were enrolled to first-line Antiretroviral treatment from January 2013 to May 2020 in Kolfe Keranyo sub-city. Data was collected by using data extraction checklist and data were extracted by reviewing children’s medical chart and electronic database. Kaplan–Meier method was used to estimate the probability of treatment failure. During bivariable analysis variables with p-value < 0.25 were taken for multivariable Cox regression analysis to assess predictors of treatment failure. Statistically significant association was declared at p-value < 0.05 with 95% confidence interval.<br><br>Result : The overall treatment failure rate within the follow-up period was 17.2%. This study also found that the overall incidence density rate was 3.45% (95% CI: 2.57-4.67) per 1000 person-month observation. Infant prophylaxis for PMTCT (AHR: 3.59, 95% CI: 1.65-7,82), drug substitution (AHR: 0.18, 95% CI: 0.09-0.37), AZT/3TC/NVP based regimen (AHR: 2.27, 95% CI: 1.14-4.25), and more than 3 episodes of poor ART adherence (AHR: 2.27, 95% CI: 1.17-4.38) were found to be predictors of treatment failure among children.<br><br>Conclusion : High proportion of treatment failure was found among children on first-line ART in Kolfe Keranyo sub-city, Addis Ababa. Infant prophylaxis for PMTCT, drug substitution, initial regimen, and poor ART adherence were found to be predictors of first-line ART treatment failure. Close follow-up of children on medication adherence and effective trainings to health care professionals need to be considered.
**Order of Authors:**	Daniel Angassa
	Meseret Misasew, MPH
**Additional Information:**	
**Question**	**Response**
**Financial Disclosure**<br><br>Enter a financial disclosure statement that	The authors have not received any finical support from any institution/government; hence, the operation of the research is solely financed by the handling fee received from the authors.

Powered by Editorial Manager® and ProduXion Manager® from Aries Systems Corporation

describes the sources of funding for the work included in this submission. Review the submission guidelines for detailed requirements. View published research articles from *PLOS ONE* for specific examples.<br><br>This statement is required for submission and **will appear in the published article** if the submission is accepted. Please make sure it is accurate.<br><br>**Unfunded studies**<br>Enter: *The author(s) received no specific funding for this work.*<br><br>**Funded studies**<br>Enter a statement with the following details:<br>• Initials of the authors who received each award<br>• Grant numbers awarded to each author<br>• The full name of each funder<br>• URL of each funder website<br>• Did the sponsors or funders play any role in the study design, data collection and analysis, decision to publish, or preparation of the manuscript?<br>• **NO** - Include this sentence at the end of your statement: *The funders had no role in study design, data collection and analysis, decision to publish, or preparation of the manuscript.*<br>• **YES** - Specify the role(s) played.<br><br>* typeset	
**Competing Interests**<br><br>Use the instructions below to enter a competing interest statement for this submission. On behalf of all authors, disclose any competing interests that could be perceived to bias this work—acknowledging all financial support and any other relevant financial or non-financial competing interests.<br><br>This statement is required for submission and **will appear in the published article** if the submission is accepted. Please make sure it is accurate and that any funding	The authors have declared that no competing interests exist.

sources listed in your Funding Information later in the submission form are also declared in your Financial Disclosure statement.<br><br>View published research articles from *PLOS ONE* for specific examples.<br><br>**NO authors have competing interests**<br><br>Enter: *The authors have declared that no competing interests exist.*<br><br>**Authors with competing interests**<br><br>Enter competing interest details beginning with this statement:<br><br>*I have read the journal's policy and the authors of this manuscript have the following competing interests: [insert competing interests here]*<br><br>* typeset	
**Ethics Statement**<br><br>Enter an ethics statement for this submission. This statement is required if the study involved:<br><br>• Human participants<br>• Human specimens or tissue<br>• Vertebrate animals or cephalopods<br>• Vertebrate embryos or tissues<br>• Field research<br><br>Write "N/A" if the submission does not require an ethics statement.<br><br>General guidance is provided below. Consult the submission guidelines for detailed instructions. **Make sure that all information entered here is included in the Methods section of the manuscript.**	Ethical clearance was obtained from the Institution Review Board (IRB) of Addis Ababa Public Health Research and Emergency Management Directorate. Then, official letter was submitted to the selected health facilities and permission was obtained to access the ART client’s database and charts. To maintain confidentiality patient name and unique ART number were not included in the data extraction checklist. Furthermore, confidentiality of data was kept at all levels of the study and not used for any other purposes than the stated study objectives.

Powered by Editorial Manager® and ProduXion Manager® from Aries Systems Corporation

**Format for specific study types**<br><br>**Human Subject Research (involving human participants and/or tissue)**<br>• Give the name of the institutional review board or ethics committee that approved the study<br>• Include the approval number and/or a statement indicating approval of this research<br>• Indicate the form of consent obtained (written/oral) or the reason that consent was not obtained (e.g. the data were analyzed anonymously)<br><br>**Animal Research (involving vertebrate animals, embryos or tissues)**<br>• Provide the name of the Institutional Animal Care and Use Committee (IACUC) or other relevant ethics board that reviewed the study protocol, and indicate whether they approved this research or granted a formal waiver of ethical approval<br>• Include an approval number if one was obtained<br>• If the study involved *non-human primates*, add *additional details* about animal welfare and steps taken to ameliorate suffering<br>• If anesthesia, euthanasia, or any kind of animal sacrifice is part of the study, include briefly which substances and/or methods were applied<br><br>**Field Research**<br><br>Include the following details if this study involves the collection of plant, animal, or other materials from a natural setting:<br>• Field permit number<br>• Name of the institution or relevant body that granted permission	
**Data Availability**<br><br>Authors are required to make all data underlying the findings described fully available, without restriction, and from the time of publication. PLOS allows rare exceptions to address legal and ethical concerns. See the PLOS Data Policy and FAQ for detailed information.	Yes - all data are fully available without restriction

A Data Availability Statement describing where the data can be found is required at submission. Your answers to this question constitute the Data Availability Statement and **will be published in the article**, if accepted.<br><br>**Important:** Stating 'data available on request from the author' is not sufficient. If your data are only available upon request, select 'No' for the first question and explain your exceptional situation in the text box.<br><br>Do the authors confirm that all data underlying the findings described in their manuscript are fully available without restriction?	
**Describe where the data may be found in full sentences. If you are copying our sample text, replace any instances of XXX with the appropriate details.**<br><br>• If the data are **held or will be held in a public repository**, include URLs, accession numbers or DOIs. If this information will only be available after acceptance, indicate this by ticking the box below. For example: *All XXX files are available from the XXX database (accession number(s) XXX, XXX.).*<br>• If the data are all contained **within the manuscript and/or Supporting Information files**, enter the following: *All relevant data are within the manuscript and its Supporting Information files.*<br>• If neither of these applies but you are able to provide **details of access elsewhere**, with or without limitations, please do so. For example:<br><br>*Data cannot be shared publicly because of [XXX]. Data are available from the XXX Institutional Data Access / Ethics Committee (contact via XXX) for researchers who meet the criteria for access to confidential data.*<br><br>*The data underlying the results presented in the study are available from (include the name of the third party*	All relevant data are within the manuscript and its Supporting Information files.

and contact information or URL).<br>• This text is appropriate if the data are owned by a third party and authors do not have permission to share the data.<br><br>* typeset	
Additional data availability information:	

Click here to access/download;Manuscript;ART_failure_Manuscript.docx

Incidence and Predictors of Antiretroviral Treatment Failure among Children in Public Health Facilities of Kolfe Keranyo Sub-City, Addis Ababa, Ethiopia: Institution-based retrospective cohort study

Meseret Misasew[1*], Daniel Angassa[2]

[1] Center for Disease Control and Prevention, Addis Ababa, Ethiopia, mesimisasew@gmail.com, Orchid ID: 0000-0001-8152-9704

[2] Lideta Sub-City Health Office, Addis Ababa, Ethiopia, danielssa25@gmail.com, Orchid ID: 0000-0001-6025-0309

[*] Corresponding author: Meseret Misasew, e-mail: mesimisasew@gmail.com

Abstract

Background: Human immunodeficiency virus (HIV) infection is a public health concern globally. The numbers of people living with HIV worldwide in 2018 was estimated at 37.9 million; from those, 1.7 million are children. Globally, 62% of the 37.9 million people were receiving Antiretroviral treatment (ART); 53% had achieved viral suppression. This study aimed to assess the incidence and predictors of Antiretroviral treatment failure among children in Kolfe Keranyo sub-city, Addis Ababa, Ethiopia.

Methods: An institution-based retrospective cohort study was conducted among 250 children who were enrolled to first-line Antiretroviral treatment from January 2013 to May 2020 in Kolfe Keranyo sub-city. Data was collected by using data extraction checklist and data were extracted by reviewing children's medical chart and electronic database. Kaplan–Meier method was used to estimate the probability of treatment failure. During bivariable analysis variables with p-value < 0.25 were taken for multivariable Cox regression analysis to assess predictors of treatment failure. Statistically significant association was declared at p-value < 0.05 with 95% confidence interval.

Result: The overall treatment failure rate within the follow-up period was 17.2%. This study also found that the overall incidence density rate was 3.45% (95% CI: 2.57-4.67) per 1000 person-month observation. Infant prophylaxis for PMTCT (AHR: 3.59, 95% CI: 1.65-7,82), drug substitution (AHR: 0.18, 95% CI: 0.09-0.37), AZT/3TC/NVP based regimen (AHR: 2.27, 95% CI: 1.14-4.25), and more than 3 episodes of poor ART adherence (AHR: 2.27, 95% CI: 1.17-4.38) were found to be predictors of treatment failure among children.

Conclusion: High proportion of treatment failure was found among children on first-line ART in Kolfe Keranyo sub-city, Addis Ababa. Infant prophylaxis for PMTCT, drug substitution, initial regimen, and poor ART adherence were found to be predictors of first-line ART treatment failure. Close follow-up of children on medication adherence and effective trainings to health care professionals need to be considered.

Introduction

Human immunodeficiency virus (HIV) infection is a public health concern globally, especially in Sub-Saharan African countries. The numbers of people living with HIV worldwide in 2018 have estimated at 37.9 million out of these 1.7 million are children. Sub-Saharan Africa remains the region most heavily affected by HIV, accounted for 67.5% of HIV infections worldwide in adults and 90% in children (1–4). New HIV infection in 2018 account 1.7 million adults and 160,000 children and 54% of children living with HIV were receiving lifelong ART in low and middle-income countries (5).

Ethiopia, to reduce the problem of HIV, began providing Antiretroviral Treatment (ART) in 2003 and free ART was launched in 2005 (1,6). According to the 2017 Ministry of Health estimate, 722,248 Ethiopians are currently living with HIV but only 420,000 are taking ART. Although ART coverage for adults has reached 61%, it remains low for children (33%) (7). In Ethiopia there is a significant pediatric HIV-1 burden with approximately 63,227 infected children and estimated 5479 new HIV infected children with 3200 AIDS-related children deaths occurring annually (1,2).

Monitoring of ART has used to ensure successful treatment, identify adherence problems, and determine whether ART regimens should be switched in case of treatment failure (2,3). Treatment failure is a suboptimal response or a lack of sustained response to treatment (8,9). Treatment failure is measured by clinical, immunological and virological failures.

The global target of a sustainable developmental goal (SDG) is to achieve 90-90-90 treatment targets at the end of 2020. These targets are first 90% of people living with HIV to know their

status, second 90% of those who know their status are to be on treatment, and third 90% of those on treatment are to be virally suppressed (2).

A systematic review and meta-analysis conducted in 2017 in Ethiopia depicted that the pooled prevalence of ART failure was estimated to 15.3% by using WHO treatment failures criteria (10). Children and adolescents are more likely to fail on ART than adults. A retrospective cohort study conducted at Addis Ababa in four hospitals, showed that 14.1% of treatment failure were in children (11).

Even though, various global and local studies indicated a high proportion of treatment failure among children (11–13), little is known about the predictors of virological failure among children in Ethiopia. Most studies define treatment failure based on the two WHO criteria, clinical and immunological criteria. Currently, routine viral load workup for monitoring treatment failure is being implemented in Ethiopia. Therefore, based on routine viral load measurement along with clinical and immunological criteria, this study aimed to assess the incidence and predictors of treatment failure among children on first line antiretroviral therapy in Kolfe Keranyo sub-city Addis Ababa, Ethiopia.

Materials and Methods

Study design, setting and population

An institution-based retrospective cohort study was conducted from January to March 2021 among children enrolled to first-line ART from January 2013 to May 2020 in selected health facilities in

Kolfe Keranyo sub-city. Kolfe Keranyo sub city is one of the eleven sub-cities of Addis Ababa, the capital city of Ethiopia. Administratively, the sub-city is divided in to 15 Woredas encompassing 1 hospital and 11 functional governmental health centers. The total population of Kolfe Keranyo sub-city in 2019 was estimated to be around 576,443. The sub-city is providing ART service to 14,161 people living with HIV. All children aged less than 15 years old who started receiving ART and are on follow-up at least for six months at the selected health facilities in Kolfe Keranyo sub-city from 2013 and 2020 were included in this study. Whereas, children with incomplete medical records were excluded (14).

Sample size and sampling technique

The required sample size was determined by using a single population proportion formula based on a similar study conducted among pediatric population in Ethiopia which showed a 17.3% prevalence of ART failure (15). An assumption of 5% margin of error, 95% confidence interval, and 80% of power were considered. Additionally, after considering a 10% non-response rate for incomplete medical records, the final sample size was 250.

The health facilities were selected based on their number of pediatric ART clients. Facilities with more than ten pediatric ART clients were selected. From the 11 health centers one hospital of in the sub-city, five health centers and one hospital were selected. After preparing a sampling frame, the total sample size was then proportionally allocated to the selected health facilities. Finally, medical charts were selected with a simple random sampling technique (**Fig. 1)**.

Data collection and quality control

A structured data abstraction checklist in English language containing the most relevant variables regarding ART treatment outcomes was adopted from different previous studies. Data were collected both from patient follow up charts and electronic database. The data abstraction checklist included socio-demographic, ART, clinical, laboratory, and treatment related characteristics. The data was retrieved by eight data clerks who have diploma in information technology and experience in managing ART data in different facilities. One data collector was assigned to collect the data from each health center and three data collectors for the hospital. A one day of training on data collection tools and process was conducted. The adopted data collection checklist was pretested to check for its completeness and consistency. The principal investigator and the assigned supervisors ensured the data completeness, accuracy and consistency.

Operational definitions

Children: Individuals with age less than 15 years old.

Virological failure: Viral load above 1000 copies/mL based on two consecutive viral load measurements in 3 months, with adherence support following the first viral load test (2).

Immunological failure: Fall of CD4 count to baseline (or below) or 50% fall from on treatment peak value or Persistent CD4 level below100 cells/mm3 (2).

Clinical failure: New or recurrent WHO stage 3 and 4 condition

Treatment failure: - Patients have clinical, immunological or virological failures.

Poor Adherence: is defined in terms of total missed appointments described by greater than three days per month (2).

Censored: Patients who completed their follow up, transferred out, or lost without developing virological failure.

Time to detection of treatment failure: The time between ART initiation and detection of treatment failure of first-line ART.

Data processing and analysis

The collected data were entered into Epi Info version 7 and analyzed using SPSS version 21. Data cleaning was performed to check for frequencies, missed or error values, and identified errors were corrected after revision of the original completed questionnaire. Descriptive statistics was used to describe socio-demographic, clinical, laboratory and treatment-related characteristics of patients. The Kaplan–Meier method was used to estimate the probability of treatment failure at different time points. Incidence of treatment failure was calculated per person months of observation. To identify the predictors of the first line treatment failure the Cox proportional hazards model was applied. First, a bivariable analysis was conducted and variables with p-value less than 0.25 were entered into a multivariable analysis to control the effects of confounders. In the multivariable analysis, variables with p-value less than 0.05 were declared as statistically significant with 95% confidence interval.

Ethical considerations

Ethical clearance was obtained from the Institution Review Board (IRB) of Addis Ababa Public Health Research and Emergency Management Directorate. Then, official letter was submitted to the selected health facilities and permission was obtained to access the ART client's database and charts. To maintain confidentiality patient name and unique ART number were not included in the data extraction checklist. Furthermore, confidentiality of data was kept at all levels of the study and not used for any other purposes than the stated study objectives.

Results

Socio-demographic and clinical characteristics

A total of 250 medical charts were included in this study with a 100% response rate. Slightly more than half (55.2%) were female while the remaining 44.8% were male. This showed that a good proportion of male and female sample was taken for the study. The mean age at start of ART was 10.5 years (SD ± 3.36) with minimum age of 1 and maximum age of 14 years. The majority of participants (58.8%) fell in the age group 10-14 years. The majority of the participants (80%) lived with at least one of their biological parents. About three quarters (76.4%) of the care givers had a positive HIV serologic status. The mean follow-up period after ART initiation was 49.2 months ranging from 6 to 95 months (**Table 1**).

Table 1. **Socio-demographic characteristics children on ART at Kolfe Keranyo sub-city, Addis Ababa, Ethiopia from 2013 to 2020 (n = 250)**

Variables		Frequency	%
Gender	Male	112	44.8%
	Female	138	55.2%
Age (years)	< 5	27	11.8%
	5-10	76	30.4%
	> 10	147	58.8%
Parent status	Both Alive	133	53.2%
	Single Alive	74	29.6%
	None of them	43	17.2%
Parent taking ART	Yes	171	68.4%
	No	33	13.2 %
	Unknown	46	18.4%
Primary Caretaker	Mother	99	39.6%
	Father	15	6%
	Both parent	86	34.4
	Brother/Sister	21	8.4%
	Other/relative	29	11.6%
Caregiver HIV status	Positive	191	76.4%
	Negative	9	3.6%
	Unknown	50	20.0%
Months on ART	<= 36	116	46.4%

	37-60	106	42.4%
	> 60	28	11.2%

Baseline clinical and antiretroviral treatment characteristics

One hundred nineteen (47.6%) of study participants were found on AZT-3TC-NVP ART regimen during ART initiation. The median baseline CD4 count was 461 cells/mm3 (IQR: 304-607). More than half (63.6%) of the study participants had experience of regimen change during their ART follow-up time when the program shifted from D4T to AZT or TDF and NNRTI to DTG or LPV/r. During their follow–up time, the majority of the study participants (88.8%) did not take PMTCT prophylaxis, even if born from HIV infected mothers. Among the children on ART, half (50.4%) took Isoniazid prophylaxis on their follow-up time (**Table 2**).

Table 2. Baseline clinical and antiretroviral treatment characteristics of children on first line ART at Kolfe Keranyo sub-city, Addis Ababa, Ethiopia from 2013 to 2020 (n = 250)

**Variables**		**Frequency**	**%**
WHO stage	Stage I	141	56.4%
	Stage II	65	26%
	Stage III	36	14.4%
	Stage IV	8	3.2%
Regimen at initiation	D4T/3TC/NVP	14	5.6%
	D4T/3TC/EFV	2	0.8%
	AZT/3TC/NVP	119	47.6%

	AZT/3TC/EFV	52	20.8%
	TDF/3TC/DTG	14	5.6%
	ABC/3TC/LPV/r	14	5.6%
	ABC/3TC/DTG	7	2.8%
	AZT/3TC/LPV/r	6	2.4%
	TDF/3TC/EFV	20	8%
Infant Prophylaxis	Yes	28	11.2%
	No	222	88.8%
Disclosure of HIV status	Disclosed	116	46.4%
	Not Disclosed	106	42.4%
	Unknown	28	11.2%
Status last visit	Alive	220	88%
	Transfer Out	15	6 %
	Dead	2	0.8%
	Drop	8	3.2%
	Lost	5	2%
Isoniazid Prophylaxis	Yes	126	50.4%
	No	124	49.6%
Regimen substitutions	Yes	159	63.6%
	No	91	36.4 %

Incidence of Treatment failure

Among the 250 study subjects, 82.8% were right censored (free of treatment failure). It was found that a total of 43 subjects (17.2%) have treatment failure. Of the failures, 62.8% were virological, 18.6% were immunological, and 2.3% were clinical failures. A mixed virological and immunological failure accounted for 16.3%. The overall incidence rate was 3.45 (95% CI: 2.57-4.67) per 1000 person-years of observations. The incidence of virological failure was 2.74 (95% CI: 1.95-3.83) per 1000 person-months, immunological failure was 1.13 (95% CI: 0.67-1.91) per 1000 person-months and clinical failure was 0.08 (95% CI: 0.01- 21.5) per 1000 person-months (**Fig. 2**).

Predictors of treatment failure

The bivariable Cox proportional hazard analysis showed that baseline WHO stage III and IV, regimen substitution, parent's HIV status, infant prophylaxis at birth, initial drug regimen, parent taking ART, and episodes of poor adherence were the significant predictors of treatment failure.

In the multivariable Cox regression analysis, infant prophylaxis, drugs substitutions, initial drug regimen, and poor adherence were the predictors of treatment failure. Accordingly, children who took infant prophylaxis at birth had about 3.6 times (AHR: 3.59, 95% CI: 1.65-7,82) higher risk towards treatment failure when compared to those who didn't took infant prophylaxis. Children with drug substitutions were by 82% less likely to suffer from treatment failure when compared to those without drug substitutions (AHR: 0.18, 95% CI: 0.09-0.37). Children who were on AZT/3TC/NVP based initial treatment regimen had about 2.3 times (AHR: 2.27, 95% CI: 1.14-

4.25) higher risk towards treatment failure when compared to those who were on other initial regimens. Moreover, children with more than 3 episodes of poor ART adherence had about 2.3 times (AHR: 2.27, 95% CI: 1.17-4.38) higher risk towards treatment failure when compare to those with less than 3 episodes of poor adherence (**Table 3**).

Table 3. Multivariable Cox regression analysis showing predictors of ART treatment failure at Kolfe Keranyo sub-city, Addis Ababa, Ethiopia from 2013 to 2020 (n = 250)

Variables	Treatment Failure		AHR (95% CI)	p-value
	No	Yes		
	n (%)	n (%)		
**Infant prophylaxis**				
No	190 (85.6)	32 (14.4)	1	
Yes	17 (60.7)	11 (39.3)	3.59 (1.65-7.82)	**0.001**[*]
**Parent receiving ART**				
No	72 (91.1)	7 (9.1)	1	
Yes	135 (78.9)	36 (21.1)	0.87 (0.21-3.56)	0.846
**Drug substitution**				
No	63 (69.2)	28 (30.8)	1	
Yes	145 (91.2)	14 (8.8)	0.19 (0.09-0.37)	**<0.001**[*]
**Baseline WHO stage**				
Stage I and II	184 (85.2)	32 (14.8)	1	
Stage III and IV	24 (70.6)	10 (29.4)	0.81 (0.37-1.78)	0.599
**Initial ART regimen**				
Other than AZT/3TC/NVP	118 (90.1)	13 (9.9)	1	
AZT/3TC/NVP	89 (74.8)	30 (24.2)	2.27 (1.14-4.55)	**0.020**[*]
**Episodes of poor adherence**				
<3 poor adherence	206 (85.1)	36 (14.9)	1	
>3 poor adherence	1 (12.5)	7 (87.5)	2.27 (1.17-4.38)	**0.015**[*]
**Parent's HIV status**				
Positive	153 (80.1)	38 (19.9)	1	
Negative	54 (91.5)	5 (8.5)	1.77 (0.36-8.59)	0.487

Discussion

Despite the availability of ART drugs, antiretroviral treatment failure has been a concerning issue globally especially in developing countries like Ethiopia (10,16). This study was conducted to assess the incidence and predictors of treatment failure among children on first line antiretroviral therapy in Kolfe Keranyo sub-city Addis Ababa, Ethiopia. In this study a 17.2% treatment failure with 3.45 (95% CI: 2.57-4.67) per 1000 person-month incidence rate were observed. Moreover, infant prophylaxis, drugs substitutions, initial drug regimen, and poor adherence were the predictors of treatment failure.

The overall treatment failure in this study was 17.2%; virological, immunological and clinical failure accounted for 79%, 18.6% and 2.3% respectively. The current finding is comparable with the finding reported at Fiche and Kuyu hospital, 18.9% (17), Gondar hospital, 18.2% (18), and a study in Ethiopia, 17.3% (15). On the other hand, treatment failure was found to be lower as compared to the findings reported in black lion hospital, 22.6% (19), Kenya, 37% (20), Uganda, 34% (9), and Zimbabwe, 30.6% (21). However, it is higher than that of the study conducted in Amhara regional state, 7.7% (8), Amhara region referral hospitals, 12.1% (1), Felegehiwot referral hospital, 10.7% (22), India, 5% (23), and Addis Ababa, 14.1% (11).

In this study, virological failure shared the highest number of failure followed by immunologic failure and only small proportion of clinical failure which is similar to the findings in Amhara region referral hospital (1), Uganda (9), and Kenya (20). The reasonfor the highest proportion of virological failure might be due to that virologic failure early detects treatment failure than immunological and clinical failure.

In this study PMTCT prophylaxis at birth was found as a predictor of treatment failure. Comparable findings was reported in studies conducted in Uganda (9) and Gondar (18). NVP is the most commonly used drugs during PMTCT and it is also used as a backbone for NNRTI regimen of HAART, which might result in a higher risk of treatment failure.

This study showed that drug substitution was about 18% protective for treatment failure. This finding contradicts with the study results in Addis Ababa (11), Gondar (18) and Felegehiwot hospital (22). This might be due to the previous use of highly mutated drugs as a substitute drug. Currently, most of substitutions are provided by newly drug available drugs (protease inhibitors and integrase inhibitors) for optimized pediatric regimen.

AZT/3TC/NVP based initial regimen was also one of the predictors of the treatment failure in this study. This is similar to the findings reported in studies conducted in Amhara region referral hospital (1) and Addis Ababa (11). Whereas, studies conducted in Uganda (9) and Zimbabwe (21) reported NVP based regimen as a predictor of treatment failure. The fact that AZT/3TC/NVP regimen is not being used currently could explain the observed variations.

Poor ART adherence among children on first-line ART was another predictor of treatment failure in this study. This finding is supported by various previous studies conducted in Ethiopia (15), Uganda (9), Gondar (18), Felegehiwot hospital (22), Amhara region referral hospital (8), and Fiche and Kuyu hospital in Oromia region(17). This could be explained by poor adherence highly contributed to mutations and resistance of antiretroviral drugs which might result in treatment failure.

Contrary to the findings in several other studies (11,17,22,24), baseline CD4 count was not a predictor of treatment failure in this study. Whereas, consistent findings were reported in studies conducted in Amhara region different hospitals (1,8). This variation could be explained by recently, ART is being provided for every HIV positive individual and baseline CD4 is not being done routinely for each and every patient. In fact, patients with a very low CD4 count are more likely to have been exposed to different OIs (opportunistic infections).

Studies conducted in Amhara region referral hospitals (1), Black lion hospital (19), and Fiche and Kuyu (17) reported that WHO clinical stages and OIs as significant predictors of treatment failure. Whereas, this study reported a contradictory finding, which is consistent with the findings reported in Uganda (9). This variation might be due to the test and treat strategy differences, there is a high public health advocacy approach to ART nowadays. Besides, the use of small sample size could also be another reason.

Moreover, HIV status disclosure and the status of primary caretaker were not predictors of treatment failure in this study. These findings were in contrast with the findings reported in Amhara region (8) and Black lion hospital (19). These variations might be due to difference in sample size. Most of the children's caretakers were their own parents, this might also be another reason for the observed variations.

Although this is retrospective cohort study, there were few incomplete documentations that can be considered as a limitation. The temporal relationship between CD4 count determinations and concurrent illnesses was also difficult to determine due to the retrospective nature of the study.

Conclusions

The proportion of treatment failure among children on first-line ART in Kolfe Keranyo sub-city in Addis Ababa was found to be high according to UNAIDs plan of 3rd 90. Poor ART adherence, Infant prophylaxis for PMTCT, drug substitution, initial regimen, and poor adherence were found to be the predictors of first-line ART treatment failure. Hence, stakeholders working on HIV need to focus on these factors in order to minimize the rate of treatment failure. Early detection of treatment failure is important for optimal management of HIV infected children receiving ART.

Parents or caregivers should strictly support children on antiretroviral treatment adherence. As most of the children are minors and did not know their HIV status, parents or caregivers should be responsible in following children on medication adherence. Similarly, health care providers should also strictly support children on medication adherence. Effective trainings to health professionals based on revised guidelines is also essential. Furthermore, future studies should examine other underlying relevant factors including viral load as a measurement for treatment failure.

Acknowledgements

We would also like to express our gratitude to Addis Ababa City Administration Health Bureau and Kolfe Keranyo sub-city for allowing us to access the required patient data. We also would like to thank the data collectors and supervisors who contributed significantly for the success of this research work.

Authors’ Contributions

Conceptualization: Meseret Misasew.

Data Curation: Meseret Misasew.

Formal Analysis: Meseret Misasew, Daniel Angassa.

Funding Acquisition: Meseret Misasew.

Investigation: Meseret Misasew, Daniel Angassa.

Methodology: Meseret Misasew, Daniel Angassa.

Project Administration: Meseret Misasew.

Resources: Meseret Misasew.

Software: Meseret Misasew.

Supervision: Meseret Misasew.

Validation: Meseret Misasew, Daniel Angassa.

Visualization: Meseret Misasew.

Writing – Original Draft Preparation: Meseret Misasew, Daniel Angassa.

Writing – Review & Editing: Meseret Misasew, Daniel Angassa.

Abbreviations

AIDS, Acquired Immune Deficiency Syndrome; ART, Antiretroviral Treatment; AZT, Zidovudine; DTG, Dolutegravir; HAART, Highly Active Antiretroviral Therapy; HIV, Human Immunodeficiency Virus; IRB, Institution Review Board; LPV/r, Lopinavir / Ritonavir; NNRTI, Non-Nucleotide Reverse Transcriptase Inhibitor; NRTI, Nucleotide Reverses Transcriptase Inhibitor; NVP, Nevirapine; OI, Opportunistic Infection; PI, Protease Inhibiter; PMTCT, Prevention of Mother to Child Transmission; SPSS, Statistical Package for Social Sciences; TB, Tuberculosis; VL, Viral Load; WHO World Health Organization; 3TC, Lamivudine.

Availability of data and materials

The datasets used and/or analyzed during the current study are available from the corresponding author on reasonable request.

Competing interests

The authors declare that they have no competing interests.

Funding

We have not received any finical support from any institution/government; hence, the operation of the research is solely financed by the handling fee received from the authors.

References

1. Yihun BA, Kibret GD, Leshargie CT. Incidence and predictors of treatment failure among children on first-line antiretroviral therapy in Amhara Region Referral Hospitals, northwest Ethiopia 2018: A retrospective study. PLoS One. 2019;14(5):1–15.

2. NATIONAL GUIDELINES FOR COMPREHENSIVE HIV PREVENTION , CARE AND TREATMENT. 2018;(February).

3. Sheet F, Day WA, People V. Global hiv statistics *24.5. 2019;(June).

4. Boerma RS, Boender TS, Sigaloff KCE, De Wit TFR, Van Hensbroek MB, Ndembi N, et al. High levels of pre-treatment HIV drug resistance and treatment failure in Nigerian children. J Int AIDS Soc. 2016;19(1):1–8.

5. Chandrasekaran P, Shet A, Srinivasan R, Sanjeeva GN, Subramanyan S, Sunderesan S, et al. Long ‐ term virological outcome in children receiving first ‐ line antiretroviral therapy. AIDS Res Ther. 2018;1–10.

6. Fekade D, Weldegebreal T, Teklu AM, Damen M, Abdella S, Baraki N, et al. Predictors of Survival among Adult Ethiopian Patients in the National ART Program at Seven University Teaching Hospitals: A Prospective Cohort Study. Ethiop J Health Sci. 2017;27:63–71.

7. Ethiopian Public Health Institute FM off H, Control U. C for D, and ICAP at Columbia University. Ethiopia Population-Based HIV Impact Assessment (EPHIA)2017-2018. Summary Sheet: Preliminary Findings. 2018;(April):4–7.

8. Sisay MM, Ayele TA, Gelaw YA, Tsegaye AT, Gelaye KA, Melak MF. Incidence and risk factors of first-line antiretroviral treatment failure among human immunodeficiency virus-infected children in Amhara regional state, Ethiopia: A retrospective follow-up study. BMJ Open. 2018;8(4):1–9.

9. Sebunya R, Musiime V, Kitaka SB, Ndeezi G. Incidence and risk factors for first line anti retroviral treatment failure among Ugandan children attending an urban HIV clinic. AIDS Res Ther. 2013;10(1):1.

10. Endalamaw A, Mekonnen M, Geremew D, Yehualashet FA, Tesera H, Habtewold TD. HIV/AIDS treatment failure and associated factors in Ethiopia: Meta-analysis. BMC Public Health. 2020;20(1):1–12.

11. Bacha T, Tilahun B, Worku A. Predictors of treatment failure and time to detection and switching in HIV-infected Ethiopian children receiving first line anti-retroviral therapy. BMC Infect Dis. 2012;12(1):1.

12. Kadima J, Patterson E, Mburu M, Blat C, Nyanduko M, Anne Bukusi E, et al. Correction: Adoption of routine virologic testing and predictors of virologic failure among HIV-infected children on antiretroviral treatment in western Kenya(PLoS ONE (2019) 13: 11 (e0200242) Doi: 10.1371/journal.pone.0200242). PLoS One. 2019;14(1):1–15.

13. Article OAL. Immu Ologic a D Cli Ical Outcomes of Childre O Haart : a Retrospective Cohort a Alysis At Jimma U Iversity Specialized Hospital. 2006;(6):75–82.

14. Addis Ababa Population 2020 (Demographics, Maps, Graphs) [Internet]. [cited 2020 May

22]. Available from: https://worldpopulationreview.com/world-cities/addis-ababa-population/

15. Getaneh AY, Yizengaw A, Likie A. Rate and Predictors of Treatment Failure among Pediatric Population Taking Highly Active Antiretroviral Therapy in Ethiopia. J AIDS HIV Treat. 2019;1(2).

16. Assemie MA, Alene M, Ketema DB, Mulatu S. Treatment failure and associated factors among first line patients on highly active antiretroviral therapy in Ethiopia : a systematic review and meta-analysis. 2019;4:1–10.

17. Yassin S, Gebretekle GB. Magnitude and predictors of antiretroviral treatment failure among HIV-infected children in Fiche and Kuyu hospitals, Oromia region, Ethiopia: a retrospective cohort study. Pharmacol Res Perspect. 2017;5(1):1–9.

18. Abiyie Z. Prevalence of antiretroviral treatment failure and associated factors in HIV infected children on antiretroviral therapy at Gondar University Hospital, retrospective cohort study. Int J Med Med Sci. 2016;8(11):125–32.

19. Haile GS, Berha AB. Predictors of treatment failure, time to switch and reasons for switching to second line antiretroviral therapy in HIV infected children receiving first line anti-retroviral therapy at a Tertiary Care Hospital in Ethiopia. BMC Pediatr. 2019;19(1):1–9.

20. Kadima J, Patterson E, Mburu M, Blat C, Nyanduko M, Bukusi EA, et al. Adoption of routine virologic testing and predictors of virologic failure among HIV- infected children

on antiretroviral treatment in western Kenya. 2018;(Ci):1–15.

21. Makadzange AT, Chimukangara B, Birri R, Gordon M. Clinical , Virologic , Immunologic Outcomes and Emerging HIV Drug Resistance Patterns in Children and Adolescents in Public ART Care in Zimbabwe. 2015;1–18.

22. Brhane BG, Nibret E, Abay GK, Nega D, Tsegay YG. Virologic Failure and its Determinant Factors among Children in First Line on Highly Active Anti Retroviral Therapy at Felegehiwot Referral Hospital, Bahir Dar, Northwest, Ethiopia: cross-sectional study. Adv Hematol Oncol Res. 2020;3(1):7–14.

23. Mukherjee A, Shah N, Singh R, Vajpayee M, Kabra SK, Lodha R. Outcome of highly active antiretroviral therapy in HIV- infected Indian children. BMC Infect Dis. 2014;14(1):1–7.

24. Getaneh Y, Egziabhier AG, Zealiyas K, Tilahun R, Girma M, Gebremedihin G. HIV-1 treatment failure among population taking Antiretroviral Therapy in Ethiopia. J AIDS HIV Treat. 2019;1(2):46–57.

 Click here to access/download;Figure;Figures.docx

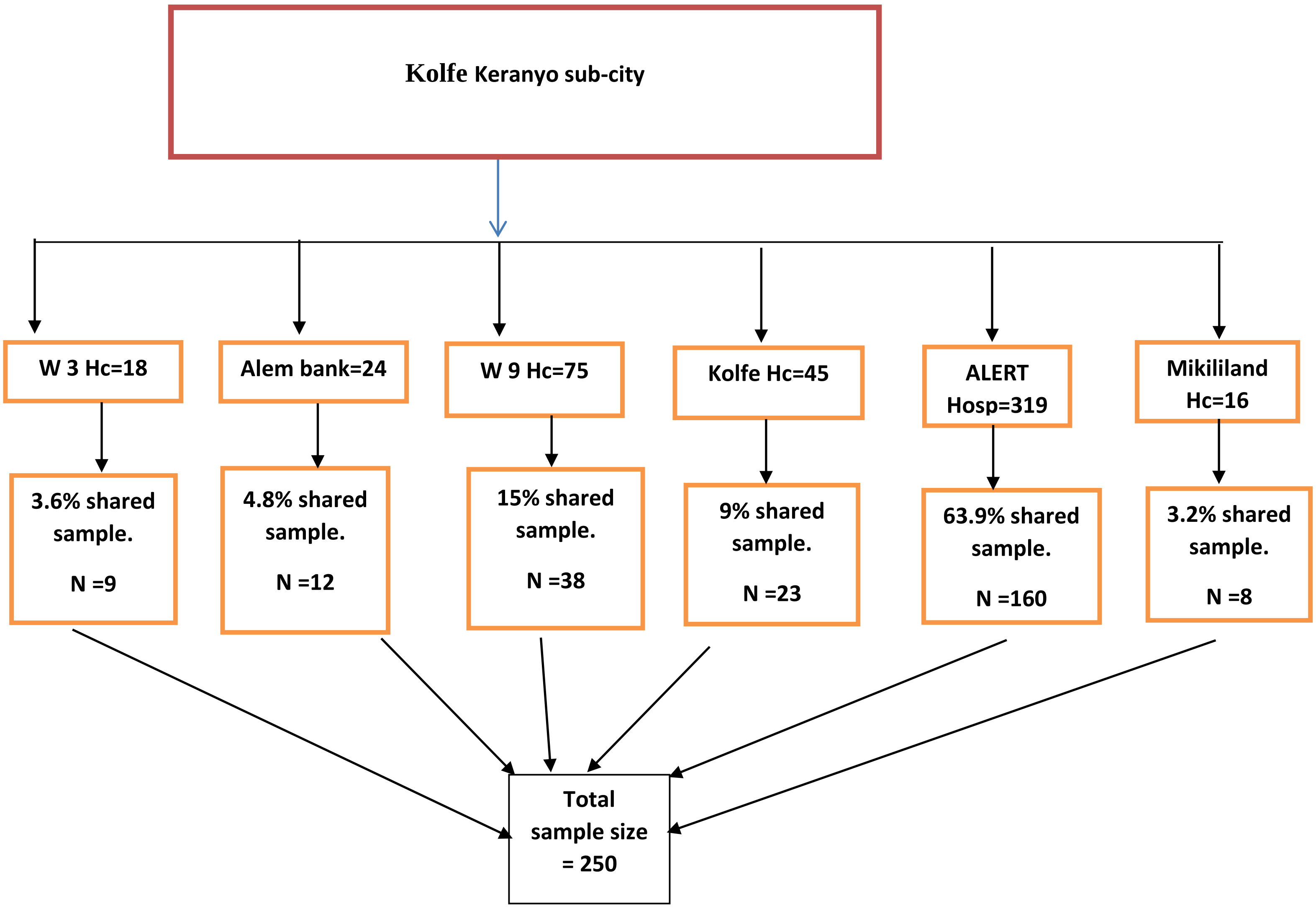

Fig. 1: Sample size distribution of study participants in Kolfe Keranyo sub-city, Addis Ababa, Ethiopia.

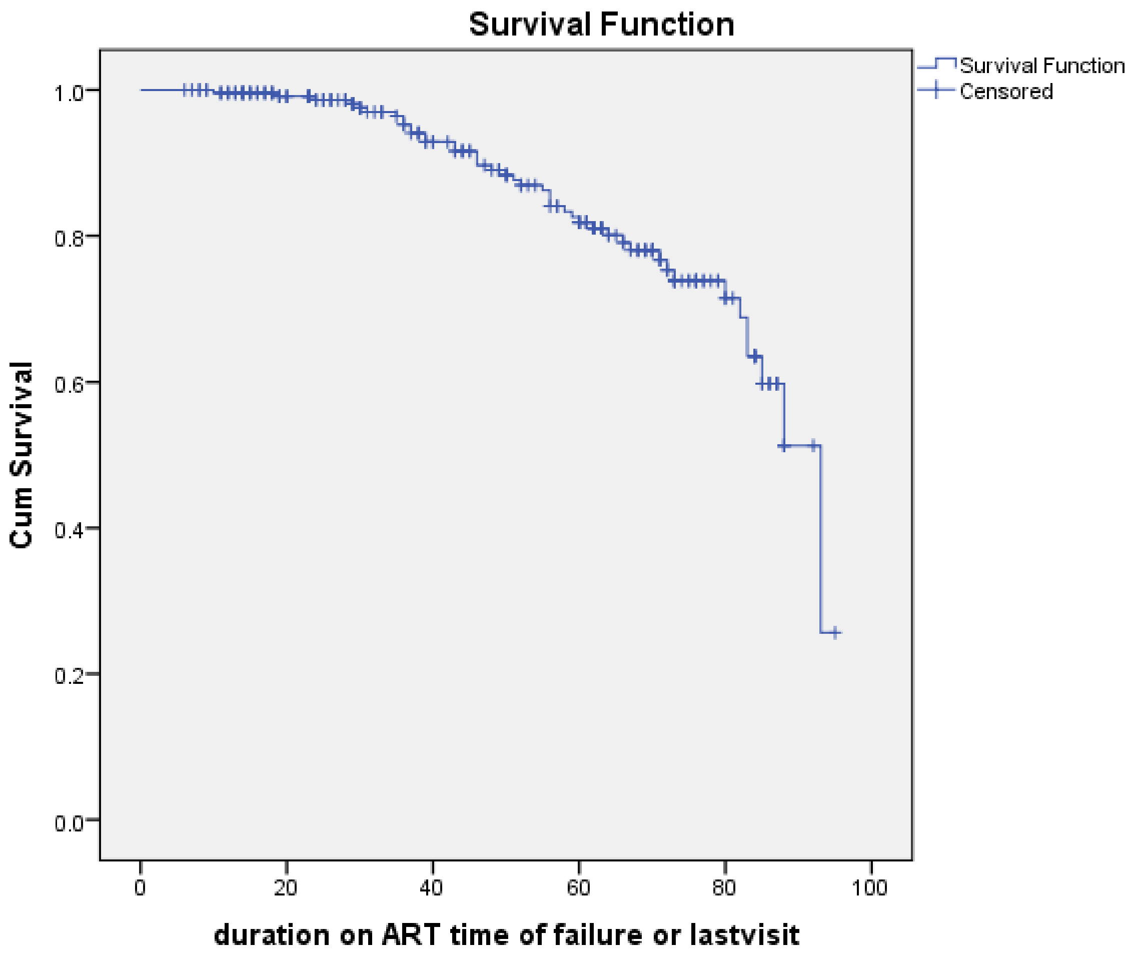

Fig. 2: The Kaplan Meier Survival function of children on first line ART at Kolfe Keranyo sub-city, Addis Ababa, Ethiopia from 2013 to 2020 (n = 250)
