# Supplementary material for: Incidence and predictors of antiretroviral treatment failure among children in public health facilities of Kolfe Keranyo Sub-City, Addis Ababa, Ethiopia: Institution-based retrospective cohort study
Source: PLoS One. 2023 Aug 18;18(8):e0266580. doi: 10.1371/journal.pone.0266580 (PMC10437829; doi:10.1371/journal.pone.0266580)
Supplement: Attachment — Submitted filename: Review_document.pdf [file pone.0266580.s005.pdf]

Title: Incidence and Predictors of Antiretroviral Treatment Failure among Children in Public Health Facilities of Kolfe Keranyo Sub-City, Addis Ababa, Ethiopia: Institution-based retrospective cohort study

PONE-D-22-08487R2

The manuscript is based on a relevant subject of ART Failure and covers an important age-group: Children (0-15 years), however, few points could be considered to improve the clarity of the manuscript.

Major observations:

- The authors may reconsider the outcome variable: Incidence/Incidence density of Treatment failure. Since the cohort of children enrolled in first-line ART were considered for the study, it could be presented as a proportion of group who developed treatment failure.
- The Incidence and Incidence density are used interchangeably in the manuscript (Abstract, Methods, Result section)
- The units for Incidence are also used interchangeably between person-months and person years (Line 141: person-months; Line 182: person-years)
- Line 279 ("Enhancing parents or caregivers and health care providers support to children on antiretroviral treatment adherence is essential for achieving the targeted viral suppression."): It will be helpful for policy makers and readers if authors add specific recommendation, as per the context and study findings. The recommendation seems general. Authors may suggest ways to enhance the support for children on ART.

Minor observations:

- Line 31: Rate is mentioned with a %. Please revise
- Line 93 ("Additionally, after considering a 10% non-response rate for incomplete medical records, the final sample size was 250."): Non-response rate are usually related to survey methods; authors should consider revising the phrase.
- Line 95 ("Facilities with more than ten pediatric ART clients were selected."): Authors must add what time period was considered for the category, 10 patients in a month/year?
- Line 156 ("A total of 250 medical charts were included in this study."): Authors must explain if each medical chart for one child or more than one medical chart were included for one child in the study.
- Line 168 ("More than half (63.6%)"): Authors must add the numbers also in the results section.
- Results section: The results must be described in the text, as per occurrence in the table.
- Line 177: Multiple phrases are used for study participants: children on ART/ medical charts/study subjects. Authors must use uniform description across the manuscript.
- Table 2: Authors may add description of category: Status last visit. Lack of clarity between the sub-category: Drop and Lost. The descriptions can be added in the Methods section, and as a footnote to the table.

END
