# Supplementary material for: Incidence and predictors of antiretroviral treatment failure among children in public health facilities of Kolfe Keranyo Sub-City, Addis Ababa, Ethiopia: Institution-based retrospective cohort study
Source: PLoS One. 2023 Aug 18;18(8):e0266580. doi: 10.1371/journal.pone.0266580 (PMC10437829; doi:10.1371/journal.pone.0266580)
Supplement: Attachment — Submitted filename: Response to Reviewers.docx [file pone.0266580.s003.docx]

**Response to Reviewers**

**Title:** Incidence and Predictors of Antiretroviral Treatment Failure among Children in Public Health Facilities of Kolfe Keranyo Sub-City, Addis Ababa, Ethiopia: Institution-based retrospective cohort study

**Authors:**

Meseret Misasew ([mesimisasew@gmail.com](mailto:mesimisasew@gmail.com))

Daniel Angassa ([danielssa25@gmail.com](mailto:danielssa25@gmail.com))

Yesunesh Teshome ([yesunesh.teshome@yahoo.com](mailto:yesunesh.teshome@yahoo.com))

**Version: 1 Date:** 15 August 2022

Response to reviewers’ comments and suggestions

We would like to thank both the editor and reviewers for taking the time to read our paper and forward their valuable comments and suggestions which in turn helped us to make a significant improvement to our manuscript. We have attempted to address all the reviewers' comments and suggestions and carefully took corrective measures. Additionally, we have changed the affiliation of one author, Daniel Angassa, and included one additional author, Yesunesh Teshome, full information including affiliation is included in the revised manuscript. These changes can be found highlighted in light blue color in the title page and Authors’ contribution section of the revised manuscript, lines 5, 8, 10, 291, 293, 294, 299, 301, and 302.

Furthermore, the point-by-point response to the editor’s and reviewers’ comments is presented below. Our responses are written in blue font color.

Academic Editor:

i. Please describe the standard treatment guidelines in the country and whether the regimen prescribed for patients in your study differed.

Authors’ response: The standard treatment guideline implemented in Ethiopia states that all HIV positives are eligible for ART. The preferred first-line regimen for children and adolescents is AZT or ABC + 3TC + EFV. The alternative first-line regimes are AZT + 3TC + NVP, TDF + 3TC + EFV, TDF + 3TC + NVP, and ABC + 3TC + NVP. The aforementioned regimes are being implemented all over the country and the regimens prescribed in our study were similar to those regimens.

ii. Isoniazid prophylaxis – is it initiation of completion of IPT? Also, how long ago was this IPT prescribed?

Authors’ response: It is the initiation of IPT and IPT was prescribed for at least six months, as the national treatment guideline recommends.

iii. Were any patients’ regimen was switched due to failure?

Authors’ response: Yes, among patients who had treatment failure, 69.8% had switched regimen to a second-line regimen. We have included this sentence and presented it in a table in the results section of the revised manuscript. These changes can be found highlighted in the results section of the revised manuscript, lines 181-182.

iv. Please proofread the manuscript and check the typos and grammar.

Authors’ response: Thank you for your valuable comment. As suggested, we proofread and made significant grammatical and technical corrections to all parts of the manuscript.

Reviewer # 1:

1. Abstract:
   i. The sentence is not very clear, this is 53% from the ones who are on ART? or from the total number of HIV infected?
   ii. Which initial regimen?

Authors’ response: Thank you for raising these questions.

i. On the selected sentence, 53% represents those who are on ART and had achieved viral suppression. We have improved the sentence in order to make it clear to readers.
ii. The initial regimen mentioned in this section is AZT/3TC/NVP based regimen and we have included it to make it more clear to readers.

These changes can be found highlighted in the abstract section of the revised manuscript, lines 19 and 38.

2. Introduction:
   i. It is also very low for adults, in this sentence seams that 61% coverage is acceptable but is not as is very far from the target of 90%.
   ii. Will be more interesting to know the % of children failing treatment.
   iii. Since when is implemented? as this study review data from 2013 to 2020 the assessment of treatment failure will be diferent before and after implementation of routine viral load.

Authors’ response: Thank you again for your valuable comments.

i. As you stated 61% ART coverage for adults is also low as compared to the target, 90%. Hence, we have improved the sentence in a way that makes more sense.
ii. In the selected sentence, 14.1% was intended to represent the percentage of children with treatment failures and we have improved the sentence in a better meaningful way.
iii. Routine viral load workup for monitoring treatment failure is being implemented in Ethiopia, since 2016. We have used viral load measurement along with clinical and immunological criteria to assess treatment failure in our study.

These changes can be found highlighted in the introduction section of the revised manuscript, lines 51-53 and 67-68.

3. Materials and Methods:
   i. To mention somewhere definition of adherence support, as structured talk with a counselor, use of reminders, or any other strategy.....
   ii. This is appointment to health center? how many appointments had the patients per month??.....miss dosages are not counted as poor adherence?

Authors’ response: Thank you again for the important suggestions.

i. We have included definitions of the terms ‘Adherence’ and ‘Adherence Support’.
ii. Since counting missed doses is the correct way of monitoring adherence, we have also changed the word ‘appointments’ into ‘doses’ on the definition of poor adherence.

These changes can be found highlighted in the materials and methods section of the revised manuscript, lines 120-123.

4. Results:
   i. Why to say during follow up time, is NVP prophylaxis is done at birth before decision of starting ART?
   ii. What is the difference between drop and lost?
   iii. Which one?

Authors’ response: Thank you again for raising these important questions.

i. NVP prophylaxis is given to all infants born to HIV infected mothers within one hour of birth before the decision of starting ART.
ii. We have included the definitions of ‘Lost’ and ‘Drop’ in the operational definitions.
iii. The mentioned initial regimen is AZT/3TC/NVP based regimen and we have included it in every section of the revised manuscript.

These changes can be found highlighted in the materials and methods, and results section of the revised manuscript, lines 130-133, 191,195, and 213.

5. Discussion
   i. According to description above is the lack of drug substitution what is a predictor of treatment failure.... the way is written is a bit confusing to me.
   ii. The 250 children had access to viral load? , if not perhaps somewhere to describe how many were assessed with viral load.
   iii. Was viral load assessed before drug substitution? otherwise the initial treatment before the substitution could be failed without knowing it.

Authors’ response: Thank you again for your valuable comments.

i. Lack of drug substitution was the predictor of treatment failure and we have improved the sentence accordingly on every section of the revised manuscript.
ii. The number of children who had access to viral load measurement was 220 (88%) and we have included this sentence on the revised manuscript.
iii. Initial viral load measurement was done before drug substitution which enabled us to differentiate which drug regimen failed.

These changes can be found highlighted in the results and discussion sections of the revised manuscript, lines 173-174, 190-191, and 213.

6. Conclusions:
   i. Same comments than before.
   ii. What factors?

Authors’ response: Thank you again for your valuable comments.

i. We accepted the comment and improved the sentence in the revised manuscript.
ii. As explained in the introduction section of the manuscript, literature regarding predictors of virological failure among children in Ethiopia is scarce. Therefore, we are suggesting future broader studies to assess other relevant factors including viral load as a measurement for treatment failure. We have amended accordingly some sentences in the conclusion section of the revised manuscript.

These changes can be found highlighted in the conclusions section of the revised manuscript, lines 274, 281, and 282.

Reviewer # 2:

i. Introduction:

It would be interesting to include a couple of sentences on why you decided to carry out this study and what you hope to achieve based on the conclusions drawn.

Authors’ response: Thank you for your valuable comment. We accepted the comment and amended the suggested sentence in the introduction section of the revised manuscript. This change can be found highlighted in the introduction section of the revised manuscript, lines 73-76.

ii. Methods:
    i. As regards ethics, please mention specifically why consent of patients was waived for this study.
    ii. Why is your sample size derived proportionally as opposed to including all participants that meet selection criteria?
    iii. Please reference the studies from which you derived your questionnaire.
    iv. How was your data anonymized and who had access to the key? Also, can you explain how you dealt with other identifying factors such as date of birth and address?
    v. Please indicate how bias was addressed. I see some sources. For example: You select centres with >10 children. This means that they are slightly larger centres, probably with more expertise. Also, I believe that the test and treat strategy was implemented in Ethiopia only after 2017, so this might bias results.
    vi. The definition of adherence is unclear. Why are missed appointments mentioned? How is adherence measured in the study?
    vii. Please define drug substitution in the operational definitions. Also, it would be good to have a reference of which drugs are used for EMTCT.
    viii. I see that you have defined the p value as 0.25 for bivariate analysis. Can you explain why you have chosen this threshold?

Authors’ response: Thank you again for your valuable comments and questions.

i. Since we have used secondary data and there was no contact with patients, the selected health facilities have signed consent and gave permission to access the ART patient’s database and charts rather than obtaining consent of patients.
ii. We have derived our sample size proportionally rather than including all participants who meet the selection criteria due to budget and resource constraints since the authors haven’t received any financial support or fund.
iii. We have cited the studies from which we adopted our questionnaire and put them in the reference.
iv. Our data was anonymized by not including patient names and unique ART numbers in the data extraction checklist and only the authors had access to the original dataset. We have included date of birth/ age but not address of patients in the data set.
v. Thank you and we have included selection bias in our limitations. We have tried to minimize selection bias by increasing the number of health facilities that were included in the sampling frame, about half of the health centers were included. The only hospital with a large number of patients was also included.
vi. As explained above we have amended the definition of adherence and rather than missed appointments we have used missed doses to measure adherence.
vii. We have included drug substitution in the operational definition as suggested. The drugs used for EMTCT are NVP and AZT.
viii. Since we sustain a shortfall of predictor variables at p-value 0.05 in the bivariate analysis, we have chosen a p-value of 0.25, a commonly used cut-off value, for variables to be included in the multivariable analysis.

These changes can be found highlighted in the materials and methods section of the revised manuscript, lines 102, 120, 121, 124, 134, 268-270.

iii. Results:
     i. Can you explain how you dealt with missing data?
     ii. I suggest you stratify by patients before and after the test and treat strategy was implemented in Ethiopia. This would be an effect modifier for the baseline CD4
     iii. I see you mention virological failure in the text of your results but I do not see it in the table. Could you include that variable to, especially since you specifically refer to it in the introduction?

Authors’ response: Thank you again for your questions and important suggestions.

i. We have encountered only a few missed data and hence, we omitted those few missed data and analyzed the remaining data.
ii. We were not able to stratify patients as such because baseline CD4 assessment was not conducted for all patients before and after the test and treat strategy was implemented. There were times when CD4 machines were not fully functional all over the country.
iii. We have included a table that presented types of treatment failure including virological failure, as suggested.

These changes can be found highlighted in the materials and methods, and results sections of the revised manuscript, lines 187-188.

iv. Discussion:
    iv. You mention poor ART adherence as a predictor of treatment failure. However, your definition mentions appointments while the other studies mentioned discuss treatment adherence. Please clarify.
    v. Please mention any biases in your limitations.
    vi. It is important to mention the generalisability of your findings. How does it add to the body of knowledge on the topic at a regional and international level?

Authors’ response: Thank you again for your important suggestions and questions.

i. We have amended the definition of adherence in the revised manuscript.
ii. We have included selection bias in our limitation.
iii. As suggested, we have included a sentence about the generalizability of our findings.

These changes can be found highlighted in the materials and methods and discussion sections of the revised manuscript, lines 120-121, and 268-270.

v. Conclusion:
   i. I would suggest that you focus on factors deemed significant by your analysis in the conclusion, otherwise you run the risk of it sounding like a generic wrap-up. Focus on important factors will lead to more of a connect between the results and the conclusion.

Authors’ response: Thank you again for your suggestion. We tried to amend the conclusion in line with the significant factors from our results, as suggested. These changes can be found highlighted in the conclusions section of the revised manuscript, lines 278-281.

vi. A last comment: I saw some grammatical and syntactical errors in the article.

Authors’ response: Thank you again and we have made significant grammatical and syntactical corrections to all parts of the manuscript.

vii. In summary, I believe this article has scientific relevance and look forward to reading it when it is published.

Authors’ response: Thank you for your kind words and we have amended every section of the manuscript based on the reviewers’ and editor’s comments and suggestions.

Kind regards,

Meseret Misasew

Corresponding author
