# Supplementary material for: Incidence and predictors of antiretroviral treatment failure among children in public health facilities of Kolfe Keranyo Sub-City, Addis Ababa, Ethiopia: Institution-based retrospective cohort study
Source: PLoS One. 2023 Aug 18;18(8):e0266580. doi: 10.1371/journal.pone.0266580 (PMC10437829; doi:10.1371/journal.pone.0266580)
Supplement: Attachment — Submitted filename: Response to Reviewers.docx [file pone.0266580.s004.docx]

**Response to Reviewers**

**Title:** Incidence and Predictors of Antiretroviral Treatment Failure among Children in Public Health Facilities of Kolfe Keranyo Sub-City, Addis Ababa, Ethiopia: Institution-based retrospective cohort study

**Authors:**

Meseret Misasew ([mesimisasew@gmail.com](mailto:mesimisasew@gmail.com))

Daniel Angassa ([danielssa25@gmail.com](mailto:danielssa25@gmail.com))

Yesunesh Teshome ([yesunesh.teshome@yahoo.com](mailto:yesunesh.teshome@yahoo.com))

**Version: 2 Date:** 3 November 2022

Response to reviewers’ comments and suggestions

We would like to thank the reviewers for taking the time to read our paper and forward their valuable comments and suggestions which in turn helped us to make a significant improvement to our manuscript. We have attempted to address all the reviewers' comments and suggestions and carefully took corrective measures.

Furthermore, the point-by-point response to the reviewers’ comments is presented below. Our responses are written in blue font color.

Reviewer # 2:

1. The manuscript is presented in an intelligible fashion, but there are many grammatical errors.

Authors’ response: Thank you for your valuable comment. We have made significant grammatical corrections to all parts of the manuscript.

2. Results from the table are repeated in the manuscript text; value of repetition debatable.

Authors’ response: Thank you again for your comment. In the manuscript text, we have tried to narrate the main /predominant results presented in the tables.

3. I still have a few concerns around phrasing:
   i. Line 156 says "A total of 250 medical charts were included in this study with a 100% response rate". To me. response rate=survey
   ii. Line 178 says: "82.8% were right censored (free of treatment failure)" --> This is not the standard definition of right censoring and censoring is defined differently earlier.
   iii. Does AHR mean adjusted Hazard Ratio? It is not expanded at first occurrence, hence unclear.
   iv. Line 234: This might be due to the previous use of highly mutated drugs as substitute drugs. --> Must rephrase; virus is mutated, not the drugs.

Authors’ response: Thank you again for the important comments and suggestions.

i. We have removed the phrase ‘with a 100% response rate’ from the mentioned sentence.
ii. We have removed the phrase ‘free of treatment failure’ from the mentioned sentence to avoid inconsistency with its operational definition.
iii. We have expanded AHR at the first occurrence to make it clear.
iv. We have rephrased the mentioned sentence as suggested.

These changes can be found highlighted in the results section of the revised manuscript, lines 157, 196, 235, and 236.

4. Line 232: "This study showed that drug substitution was about 18% protective for treatment failure" --> Please check this, I think it is more that there is a 5-fold risk of failure at every point in the study if drug substitution does not occur.

Authors’ response: Thank you again for raising this important suggestion. We have rephrased the mentioned sentence as suggested. This change can be found highlighted in the results section of the revised manuscript, lines 233-234.

Reviewer # 3:

1. The clarification about ART coverage in line 51-53 still confuse. Need to be specified the source of 90% target, UNAIDS target. E.g. According to the 2017 Ministry of Health estimate, 722,248 Ethiopians are currently living with HIV but with a low ART coverage of 61% for adults and 33% for children, far from the UNAIDS target of 90%.

Authors’ response: Thank you for your valuable comment. We accepted the comment and amended the suggested sentence in the introduction section of the revised manuscript. This change can be found highlighted in the introduction section of the revised manuscript, lines 51-53.

2. Treatment failure in lines 59 is classified in so called later in the table as types, but in the text said is "measured" which doesn't seems accurate. And later refers to WHO criteria (line 71). May be harmonize IN one definition, classification and/or criteria.

Authors’ response: Thank you again for your valuable comment. We amended the mentioned sentence to make it consistent with the definitions we have used in the operational definition. We have used the standard WHO definitions for all types of treatment failures as mentioned in our operational definition. This change can be found highlighted in the introduction section of the revised manuscript, line 59.

3. "This showed that a good proportion of male and female sample was taken for the study", Do you mean that both genders are equally represented? "Good proportion" is not an adequate vocabulary.

Authors’ response: Thank you again for your valuable comment. We amended the mentioned sentence as suggested. This change can be found highlighted in the results section of the revised manuscript, line 157.

4. "One hundred nineteen (47.6%) of study participants were found on AZT-3TC-NVP ART regimen during ART initiation":...were initiated on xxxx regimen"

Authors’ response: Thank you again for your valuable comment. We amended the mentioned sentence as suggested. This change can be found highlighted in the results section of the revised manuscript, line 167.

5. Do you have the CD4 %? Being many U5 children, these seems important to be reported.

Authors’ response: Thank you again for your important suggestion and we completely agree that it is important to report DC4 % for the under 5 children. However, we were not able to report the CD4 % for under 5 children since the CD4 count was not available for each patient.

6. "During their follow–up time, the majority of the study participants (88.8%) did not take PMTCT prophylaxis, even if born to HIV infected mothers". This refers to the patient follow-up? And applies to ones who were recruited immediately after born becasue if not how to value the PMTCT prophlxis? I'd suggest to say: "among total....xxx% received PMTCT/infant prophylaxis"

Authors’ response: Thank you again for your important suggestion. We rephrased the mentioned sentence as suggested. This change can be found highlighted in the results section of the revised manuscript, lines 170-171.

7. 189-Predictors of treatment failure: there is a confusion with "regimen substitution" (line 191) with "drug substitution" (line 194. And when is talking about "drug substitution" is the shifts that Programme did d4T to AZT, EFV to DTG, etc? It will be good also to clarify if these shifts were done using VL previous shifting or not.

Authors’ response: Thank you again for your valuable comment. We amended the mentioned sentence as suggested. This change can be found highlighted in the results section of the revised manuscript, line 190.

8. For Poor Adherence definition, please clarify is the missed doses are self-reported, by pill count or which other method.

Authors’ response: Thank you again for your important suggestion. We amended the mentioned sentence as suggested. This change can be found highlighted in the materials and methods section of the revised manuscript, line 125.

9. "The reason for the highest proportion of virological failure might be that virologic failure early detects treatment failure than immunological and clinical failure." And in many places is related to the lack of access to VL.

Authors’ response: Thank you again for your comment. Lack of access to viral load was a problem previously. As mentioned in our introduction, lines 71-72, currently, routine viral load workup for monitoring treatment failure is being implemented in Ethiopia.

10. "NVP is the most commonly used drug during PMTCT and it is also used as a backbone for NNRTI regimen (of HAART-I'd take out this) which might result in a higher risk of treatment failure." Please elaborate more this idea.

Authors’ response: Thank you again for your important suggestion. We amended the mentioned sentence in a more descriptive form. This change can be found highlighted in the discussion section of the revised manuscript, lines 231-232.

11. "This study showed that drug substitution was about 18% protective for treatment failure" I don't understand this statement.

Authors’ response: Thank you again for your important comment. We have rephrased the mentioned sentence as suggested. This change can be found highlighted in the discussion section of the revised manuscript, lines 233-234.

12. "The fact that AZT/3TC/NVP regimen is not being used currently could explain the observed variations". Which variations

Authors’ response: Thank you again for your valuable comment. We have corrected the mentioned sentence. This change can be found highlighted in the discussion section of the revised manuscript, lines 241-242.

13. "This variation could be explained by recently, ART is being provided for every HIV positive individual and baseline CD4 is not being done routinely for each patient". This can explain lack of data but not lack of association

Authors’ response: Thank you again for your valuable comment. We have rephrased the mentioned sentence in a more meaningful way. This change can be found highlighted in the discussion section of the revised manuscript, lines 252-253.

14. Line 252-259: these paragraph need to be rephrase. And not clear how T&T is linked with no having relation between low CD4 and failure

Authors’ response: Thank you again for your valuable comment. We have rephrased the mentioned paragraph to make it clearer. This change can be found highlighted in the discussion section of the revised manuscript, lines 257-259.

15. "Despite these limitations, the findings of this study could 269 be a great input for the national ART treatment program" I'd suggest : Could inform natioanal HIV Programme for better practices and policies in pediatric HIV care.

Authors’ response: Thank you again for your important suggestion. We have rephrased the mentioned sentence as suggested. This change can be found highlighted in the discussion section of the revised manuscript, lines 270-271.

16. Line 273: UNAIDS virological supression targets

Authors’ response: Thank you again for your important suggestion. We have rephrased the mentioned sentence as suggested. This change can be found highlighted in the conclusions section of the revised manuscript, line 274.

17. "Health care providers should provide a timely drug substitution when required". This is applicable for treatment/regimen change when failure is detected which is different from the "drug substitution" you defined as punctual replacement of one drug becasue programmatic issues

Authors’ response: Thank you again for your valuable comment. We have omitted the sentence about drug substitution since it is associated with programmatic issues.

18. In general the conclusions need to be described in a more objective way, not as an advise for parents/stakeholders/programme

Authors’ response: Thank you again for your significant comment. We have rephrased the conclusion in a more objective way as suggested. These changes can be found highlighted in the conclusions section of the revised manuscript, lines 279-282.

Kind regards,

Meseret Misasew

Corresponding author
