# Supplementary material for: Incidence and predictors of antiretroviral treatment failure among children in public health facilities of Kolfe Keranyo Sub-City, Addis Ababa, Ethiopia: Institution-based retrospective cohort study
Source: PLoS One. 2023 Aug 18;18(8):e0266580. doi: 10.1371/journal.pone.0266580 (PMC10437829; doi:10.1371/journal.pone.0266580)
Supplement: Attachment — Submitted filename: Response to Reviewers.docx [file pone.0266580.s006.docx]

**Response to Reviewers**

**Title:** Incidence and Predictors of Antiretroviral Treatment Failure among Children in Public Health Facilities of Kolfe Keranyo Sub-City, Addis Ababa, Ethiopia: Institution-based retrospective cohort study

**Authors:**

Meseret Misasew ([mesimisasew@gmail.com](mailto:mesimisasew@gmail.com))

Takele Menna ([takele.menna@sphmmc.edu.et](mailto:takele.menna@sphmmc.edu.et))

Eyoel Berhanu ([eyoel.berhanu@sphmmc.edu.et](mailto:eyoel.berhanu@sphmmc.edu.et))

Daniel Angassa ([danielssa25@gmail.com](mailto:danielssa25@gmail.com))

Yesunesh Teshome ([yesunesh.teshome@yahoo.com](mailto:yesunesh.teshome@yahoo.com))

**Version: 3 Date:** 12 April 2023

Response to reviewers’ comments and suggestions

We would like to thank the reviewers for taking the time to read our paper and forward their valuable comments and suggestions which in turn helped us to make a significant improvement to our manuscript. We have attempted to address all the reviewers' comments and suggestions and carefully took corrective measures. Additionally, we have included two additional authors, Takele Menna and Eyoel Berhanu, full information including affiliation is included in the revised manuscript. These changes can be found highlighted in light blue color in the title page and Authors’ contribution section of the revised manuscript, lines 5, 8, 9, 10, 11, 297, 300, 302, 308, 311, and 313.

Furthermore, the point-by-point response to the reviewers’ comments is presented below. Our responses are written in blue font color.

Academic Editor:

1. Lines 50-51: Please reconsider the use of, “to reduce the problem of HIV”

Authors’ response: Thank you for your important suggestion. We have rephrased the mentioned phrase as suggested. This change can be found highlighted in the introduction section of the revised manuscript, line 52.

2. Line 52: “Ethiopians are currently” is not the correct tense

Authors’ response: Thank you for your valuable comment. We have corrected the tense of the mentioned sentence as suggested. This change can be found highlighted in the introduction section of the revised manuscript, line 54.

3. Line 54: Please rephrase “infected children” this phrase. Look at the UNAIDS guidance on terminology at <https://www.unaids.org/en/resources/documents/2015/2015_terminology_guidelines>

Authors’ response: Thank you for your valuable comment. We have rephrased the mentioned phrase as suggested. This change can be found highlighted in the introduction section of the revised manuscript, lines 56 and 57.

4. Lines 60-61: The UNAIDS goals are now 95-95-95 and not 90-90-90

Authors’ response: Thank you for your important update. We have corrected the UNAIDS target as suggested. This change can be found highlighted in the introduction section of the revised manuscript, line 62.

5. Lines 64-65: Please specify which WHO treatment failure criteria – clinical, immunological, or virological

Authors’ response: Thank you for your important suggestion. We have specified the mentioned sentence as suggested. This change can be found highlighted in the introduction section of the revised manuscript, line 67.

6. Line 156: How many charts were excluded and why?

Authors’ response: Thank you for your important question. No charts were excluded and all of the children who were enrolled in first-line Antiretroviral treatment during the study period were included.

7. After reading “This showed that almost an equivalent proportion of male and female sample as taken for the study”, it feels like you have purposively taken this proportion.

Authors’ response: Thank you for raising your vital concern. We inadvertently obtained an almost proportional sample size, without intending to do so.

8. Table 2: In Status last visit, please clarify what are “drop” and “lost”

Authors’ response: Thank you for your important suggestion. We have provided a footnote on Table 2 as suggested. This change can be found highlighted in the results section of the revised manuscript, lines 177-179.

9. Hazard ratios are not interpreted as “times higher/lower risk”. Look at PMID: 15273082

Authors’ response: Thank you for your important suggestion. As our study does not involve a clinical trial, we have interpreted the hazard ratios in a manner that aligns with the interpretation methods of most of similar studies including our references.

Reviewer:

Major Observations:

1. The authors may reconsider the outcome variable: Incidence/Incidence density of Treatment

failure. Since the cohort of children enrolled in first-line ART were considered for the study,

it could be presented as a proportion of group who developed treatment failure.

Authors’ response: Thank you for your valuable suggestion. We have ensured consistency throughout the manuscript by designating 'incidence' as the outcome variable in all sections.

2. The Incidence and Incidence density are used interchangeably in the manuscript (Abstract,

Methods, Result section)

Authors’ response: Thank you again for your valuable comment. We have amended and ensured consistency throughout the manuscript by using the word 'incidence' instead of ‘incidence density’ in all sections. This change can be found highlighted in the abstract section of the revised manuscript, line 33.

3. The units for Incidence are also used interchangeably between person-months and person

years (Line 141: person-months; Line 182: person-years)

Authors’ response: Thank you again for your valuable comment. We have amended and ensured consistency throughout the manuscript by using the unit 'person-months' instead of ‘person-years’ in all sections. This change can be found highlighted in the results section of the revised manuscript, line 186.

4. Line 279 (“Enhancing parents or caregivers and health care providers support to children on

antiretroviral treatment adherence is essential for achieving the targeted viral suppression.”): It will be helpful for policy makers and readers if authors add specific recommendation, as per the context and study findings. The recommendation seems general. Authors may suggest ways to enhance the support for children on ART.

Authors’ response: Thank you again for your valuable comment. We have amended the mentioned sentence as suggested. This change can be found highlighted in the conclusions section of the revised manuscript, lines 283-285.

Minor Observations:

1. Line 31: Rate is mentioned with a %. Please revise

Authors’ response: Thank you for your important suggestion. We have amended the mentioned word accordingly. This change can be found highlighted in the abstract section of the revised manuscript, line 33.

2. Line 93 (“Additionally, after considering a 10% non-response rate for incomplete medical

records, the final sample size was 250.”): Non-response rate are usually related to survey

methods; authors should consider revising the phrase.

Authors’ response: Thank you again for your important suggestion. We have amended the mentioned sentence as suggested. This change can be found highlighted in the methods section of the revised manuscript, line 95.

3. Line 95 (“Facilities with more than ten pediatric ART clients were selected.”): Authors must

add what time period was considered for the category, 10 patients in a month/year?.

Authors’ response: Thank you again for your valuable comment. We amended the mentioned sentence as suggested. This change can be found highlighted in the methods section of the revised manuscript, line 97.

4. Line 156 (”A total of 250 medical charts were included in this study.”): Authors must explain

if each medical chart for one child or more than one medical chart were included for one

child in the study.

Authors’ response: Thank you again for your important suggestion. We amended the mentioned sentence as suggested. This change can be found highlighted in the results section of the revised manuscript, line 157.

5. Line 168 (”More than half (63.6%)”): Authors must add the numbers also in the results section..

Authors’ response: Thank you again for your suggestion. We tried to simplify the results description to ensure its easily understandable for readers.

6. Results section: The results must be described in the text, as per occurrence in the table.

Authors’ response: Thank you again for your important suggestion. We tried to make the results description in accordance with the corresponding tables.

7. Line 177: Multiple phrases are used for study participants: children on ART/ medical charts/study subjects. Authors must use uniform description across the manuscript.

Authors’ response: Thank you again for your important comment. We have amended and ensured consistency of the mentioned words throughout the entire manuscript.

8. Table 2: Authors may add description of category: Status last visit. Lack of clarity between

the sub-category: Drop and Lost. The descriptions can be added in the Methods section, and

as a footnote to the table.

Authors’ response: Thank you again for your valuable comment. We have accepted your comment and add the footnote on Table 2. This change can be found highlighted in the results section of the revised manuscript, lines 177-179.

Kind regards,

Meseret Misasew

Corresponding author
